# A Smart Capacitive Sensor Skin with Embedded Data Quality Indication for Enhanced Safety in Human–Robot Interaction

**DOI:** 10.3390/s21217210

**Published:** 2021-10-29

**Authors:** Christoph Scholl, Andreas Tobola, Klaus Ludwig, Dario Zanca, Bjoern M. Eskofier

**Affiliations:** 1 Siemens AG, Technology, Guenther-Scharowsky-Str. 1, 91058 Erlangen, Germany; andreas.tobola@siemens.com (A.T.); klaus.ludwig@siemens.com (K.L.); 2 Machine Learning and Data Analytics Lab, Department Artificial Intelligence in Biomedical Engineering (AIBE), Friedrich-Alexander-Universität Erlangen-Nürnberg (FAU), Carl-Thiersch-Straße 2b, 91052 Erlangen, Germany; dario.zanca@fau.de (D.Z.); bjoern.eskofier@fau.de (B.M.E.); 3 Institute of Electronics Engineering, Faculty of Engineering, Friedrich-Alexander-Universität Erlangen-Nürnberg (FAU), Cauerstr. 9, 91054 Erlangen, Germany; 4 Faculty of Electrical Engineering, Precision Engineering, Information Technology, Nuremberg Institute of Technology, Wassertorstraße 10, 90489 Nürnberg, Germany

**Keywords:** capacitive sensor system, smart sensors, human–robot interaction, data quality, signal processing, embedded AI

## Abstract

Smart sensors are an integral part of the Fourth Industrial Revolution and are widely used to add safety measures to human–robot interaction applications. With the advancement of machine learning methods in resource-constrained environments, smart sensor systems have become increasingly powerful. As more data-driven approaches are deployed on the sensors, it is of growing importance to monitor data quality at all times of system operation. We introduce a smart capacitive sensor system with an embedded data quality monitoring algorithm to enhance the safety of human–robot interaction scenarios. The smart capacitive skin sensor is capable of detecting the distance and angle of objects nearby by utilizing consumer-grade sensor electronics. To further acknowledge the safety aspect of the sensor, a dedicated layer to monitor data quality in real-time is added to the embedded software of the sensor. Two learning algorithms are used to implement the sensor functionality: (1) a fully connected neural network to infer the position and angle of objects nearby and (2) a one-class SVM to account for the data quality assessment based on out-of-distribution detection. We show that the sensor performs well under normal operating conditions within a range of 200 mm and also detects abnormal operating conditions in terms of poor data quality successfully. A mean absolute distance error of 11.6 mm was achieved without data quality indication. The overall performance of the sensor system could be further improved to 7.5 mm by monitoring the data quality, adding an additional layer of safety for human–robot interaction.

## 1. Introduction

Sensor systems are intrinsic to all fields of industrial applications. With the advancement of the Fourth Industrial Revolution and the Internet of things (IoT), the monitoring of processes and devices is of growing importance. Various types of sensor systems have been developed to enhance the functionality and safety of human–robot interaction (HRI) scenarios, such as cameras, tactile sensors, capacitive sensors, or microphones [1]. As sensor systems become more powerful and as more machine learning methods are used on the sensor level, it is also necessary to monitor the results of these methods in terms of data quality.

In human–robot interaction, active and passive safety measures [2] can be added to ensure safe cooperation. According to Robla-Gomez et al. [2], passive safety measures include lightweight structures or mechanical compliance systems such as series elastic actuators [3]. Seriani et al. [4] developed a comprehensive framework for the classification of n-DOF preloaded structures as a passive safety measure to mitigate collisions in cobots. Active safety measures are characterized by actively manipulating a robot’s movement in case a potential hazardous situation is detected, requiring a way of sensing the interaction between a robot and a human. An approach has been proposed by Haddadin et al. [5], who evaluate reactive control strategies to ensure the safety of humans interacting with the robot. Kim et al. [6] developed a multi-axis force sensing method which is directly mounted on a robot cover to measure contact forces in case of collision. A combination of passive and active safety measures was introduced by Heng et al. [7]. They developed a fluid-driven soft cobot-skin, which combines passive safety measures by controlling the stiffness and active safety measures by measuring the contact force.

Capacitive sensor systems have been used to equip robots with perception capabilities, using the electrical properties of conductive objects to measure physical quantities. Hoffmann et al. [8] developed an environment-aware capacitive sensor and used a nearest neighbor search to estimate the distance to obstacles based on an environment model. Poeppel et al. [9] used a neural network to compensate external influences on capacitive sensors for robust distance estimation. Erickson et al. [10] used a neural network to estimate the relative position and pitch and yaw from a series of capacitive sensor readings. The computations mostly are offloaded to powerful machines, which involves the transmission of raw sensor data and introduces an overhead by using the communication interface. Another approach to detect the distance and gestures of a human hand is to use IQ (In-Phase-and-Quadrature) demodulation [11]. However, their approach requires a FPGA (field programmable gate array)-based controller to operate at frequencies up to 20 MHz. Other approaches include computer vision-based active safety measures, which have been summarized by Cebollada et al. [12] in an extensive review article with a focus on mobile robotics or tactile sensors [13].

Available sensor concepts often neglect the possibility of low data quality, such as outliers, bias, or drift, resulting in poor decision making or even false decisions [14]. As capacitive sensor systems are often used in safety relevant applications, such as human–robot interaction [1,2], it is crucial to monitor the data quality. The necessity to observe and track data quality for sensor systems has been recognized by various authors. Ibarguengoytia et al. [15] used Bayesian networks for real-time validation in distributed sensor systems. Bisdikian et al. [16] introduced an application-agnostic data quality model with the quality attributes of accuracy, integrity and timeliness and showed its applicability in use cases in defense and the military. Wu et al. [17] introduced the cognitive Internet of Things and defined various levels of quality monitoring, such as the quality of data and quality of information. A possible approach to perform data quality indication is out-of-distribution (OOD) detection, which refers the task of detecting whether samples differ from a known data distribution. This task is closely related to anomaly or outlier detection [18] and is classified as an unsupervised learning problem. Support vector machines (SVM) are a popular machine learning method and can also be applied to solve OOD applications, which have also previously been applied in the domain of data quality indication. Zhang et al. [19] used a hyper-ellipsoidal one-class SVM as an outlier detection method to monitor data quality in wireless sensor systems. They use this SVM variant for unsupervised outlier detection. The authors also present an analysis of computational and memory complexity. An approach using supervised machine learning methods for data quality indication was presented by Rahman et al. [20]. The authors use an ensemble of classifiers and a small amount of labeled data to predict class membership at discrete levels of data quality. However, classifier ensembles can be computationally very heavy, and the authors do not study this aspect in their work. Secondly, labeling data is expensive and prone to errors. Common capacitive sensor systems are typically equipped with microcontrollers with limited code and data memory and operating frequencies in the mid to low MHz range, such as Arduino [9] or Teensy [10]. To maintain a constant execution time of the system, it is important to keep the computational cost as low as possible. For embedded classification systems, an approach to estimate the computational cost has been studied by Jensen et al. [21]. Their approach enables the quantification of the computational cost before classification models are deployed on a target system.

Related works on capacitive sensor systems for human–robot interaction often do not consider the possibility of poor data quality. Additionally, methods are rarely studied with respect to their computational complexity. We address these issues, and the contributions of this work are as follows:

- A novel capacitive sensor skin that is capable of detecting a conductive obstacle at arbitrary angles and distances up to 200 mm;
- A system architecture with a dedicated data quality indication layer based on out-of-distribution detection as an active safety measure for HRI applications;
- A comparative study of different OOD algorithms for data quality indication by utilizing regression metrics, binary classification metrics and computational cost for evaluation;
- The quantification of computational cost in terms of memory usage and execution time;
- The study of computational cost and its influence on the sensor output and the number of false alarms associated to a given model.

## 2. Materials and Methods

We designed a capacitive sensor skin to measure the distance and angle to an object near to the sensor. The device uses a data-driven approach to map sensor readings to the target coordinates and a second algorithm to monitor data quality. The sensor detects objects at distances of less than 200 mm and assigns each predicted value an additional parameter indicating data quality. We solve the regression problem to predict the distance and angle based on sensor readings with a fully connected neural network. Secondly, we evaluate different algorithms to monitor the sensor data quality. Specifically, we evaluate one-class SVMs of different complexities and compare them to a threshold and a distance-based approach, which is inspired by the k-nearest neighbors algorithm (k-NN). The developed neural network and one SVM variant are deployed on the sensor system, where the algorithms run in an environment with tight timing and memory requirements.

### 2.1. Smart Capacitive Sensor Skin

**Figure 1:**
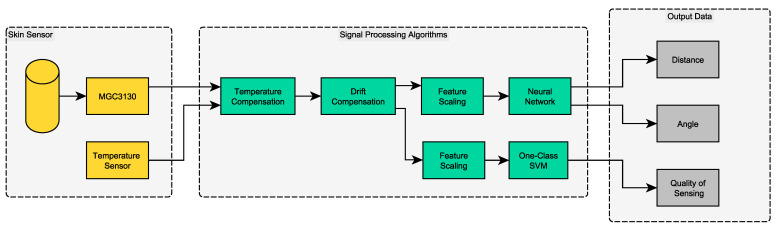
System architecture and signal processing diagram for data fusion and data quality indication. The sensor skin provides the measured physical properties as digital values to the system. Yellow blocks indicate physical parts, green blocks are algorithms implemented in software, gray blocks are signal sinks.

The basic system architecture of the smart capacitive sensor skin is displayed in Figure 1 as a block diagram. It illustrates the signal flow, starting from the capacitive sensing elements, through the signal processing algorithms to the signal sinks, where the data can be further processed by the sensor user.

**Figure 2:**
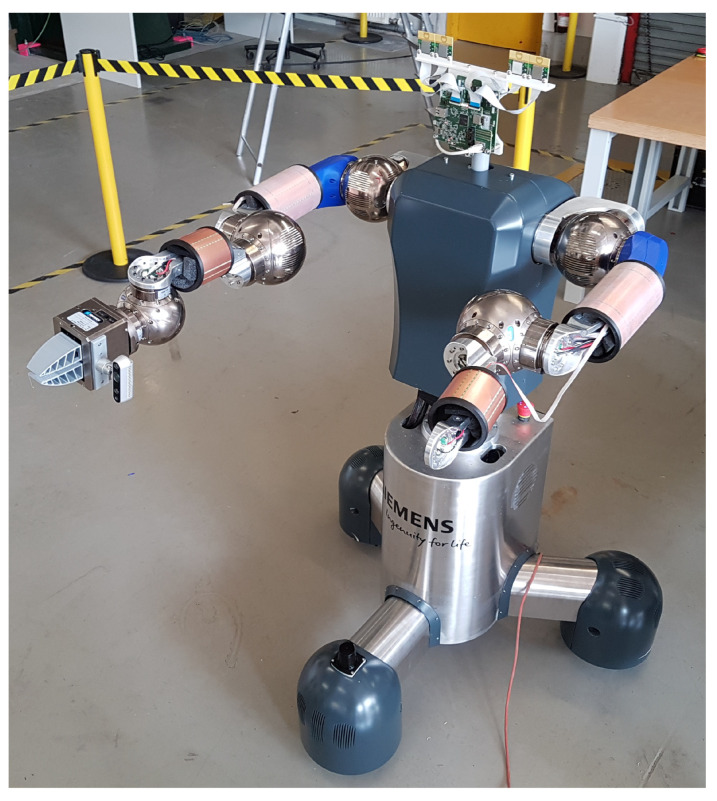
Smart capacitive sensor skin mounted on a robot. Two skin sensors are mounted on the lower arm, two on the upper arm respectively.

The sensor system is built of parts from the analog and digital domains. The copper-layered skin forms capacitors that belong to the analog domain. The sensor system is displayed in Figure 2, where it is mounted on a robot arm. This robot is meant to assist humans in heavy lifting tasks, where the capacitive skin sensor helps to avoid collisions. The sensor system is built based on a custom-designed flexible printed circuit board (PCB) on the outside that forms the top plate of the capacitive sensing device. This layer is followed by a 3D printed spacer and a copper layer, which serves as the ground plane. The mechanical design of the spacer is inspired by a honeycomb structure. The outer and inner capacitor plates are glued on the spacer to ensure proper mechanical connection. The whole sensor is covered with a shrinking tube to protect it against mechanical influences. A ribbon cable is soldered to the top plate to connect the capacitors with the PCB. The setup is a robust capacitive proximity sensor that is capable of detecting obstacles and humans nearby within a 360° radius.

As a bridge to the digital part of the system, a capacitive sensor circuit (Microchip MGC3130 [22]) is used. This chip uses electrical near-field sensing to recognize objects in the proximity of the capacitor to which it is connected. On the analog signal processing side, the chip uses so-called transmit and receive channels to measure changes in capacity. There are a total of five receive channels, called RX0 to RX4, and one transmit channel, which is named TX. The sensor signals from the RX channels are acquired using the CIC (Cascaded Integrator Comb [23]) output signal. The CIC values are given in digits and represent samples acquired from the MGC3130 analog front end by analog to digital conversion. The digital interface to the host microcontroller STM32L4 is implemented based on the Inter-Integrated Circuit (I2C) protocol for data exchange. Configuration parameters can be written to the device, and sensor values can be read from it through this interface. The used microcontroller STMicroelectronics STM32L452RET6 [24] has 512 kB of flash memory for program storage, and 160 kB of RAM. It is also equipped with a single-precision floating-point unit (FPU), which allows the fast computation of numerical algorithms. The system uses a Universal Asynchronous Receiver Transmitter (UART) interface to transmit sensor data and receive configuration parameters. In addition, two dedicated digital output pins of the microcontroller are used to signal emergencies such as “distance too low” or “insufficient data quality” instantly to sensor users. Furthermore, an external temperature sensor measures the device temperature, which is also available to the system as an input value. The sensor system runs on FreeRTOS, which is an open source licensed real-time operating system (RTOS). The operating system accounts for the specified timing demands of sampling from the sensor each 5 ms and predicting new distance, angle and data quality values for this sample within the same time frame.

### 2.2. Sensor Data Processing Workflow

To ensure a high quality of sensing (QoS) in real time, we propose the signal processing workflow illustrated in Figure 1. New measurements acquired from the sensors are preprocessed in a temperature compensation module. This module applies a linear temperature compensation model to the data to counterbalance the influence of temperature on the sensor signal. Temperature compensation is performed based on the read CIC values and the sensor temperature in Celsius. Secondly, the drift compensation module processes the data. The purpose of this block is to account for different offset levels between individual sensor devices. The individual offset is calibrated at startup when there is no object close to the sensor. It is treated as an additional bias which is offset by the temperature-compensated raw data in the drift compensation module. The compensated raw data is then scaled for prediction in the neural network to infer the target output values distance and angle. Scaling is performed by removing the population mean and population standard deviation from the temperature and drift-compensated sample. The mean and standard deviation are calculated for every CIC channel individually based on the used training data set. The same new data sample is also processed by the quality of sensing algorithm to account for the data quality indication. This data quality indication module is equipped with a separate feature scaling block to account for the properties of the used algorithm. The system provides three new output values for each sensor data sample, allowing us to track the data quality at every time step. The proposed setup imposes strict requirements on the computational requirements of the used algorithms. Depending on the required timing constraints, all used algorithms need to be lightweight enough to be computed within the given time budget. The defined time budget for the smart capacitive sensor skin is 5 ms. Secondly, the memory constraints of the computing device add further limitations.

### 2.3. Sensor Test Bench and Experimental Setup

**Figure 3:**
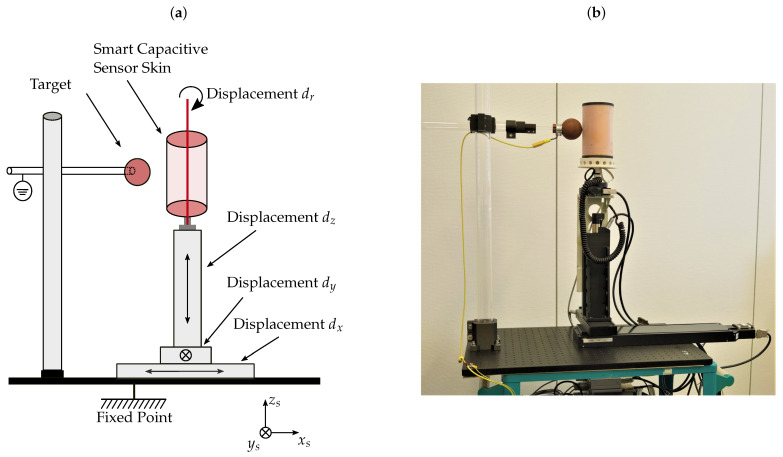
Drawing of experimental setup with coordinate system (**a**). Picture of test bench with sensor mounted (**b**).

To acquire data for training and validation purposes, a test bench was designed specifically for the needs of the smart capacitive sensor skin. The purpose of this test bench is to generate training data and to validate the developed algorithms. The test bench is displayed in Figure 3a in a schematic drawing and in Figure 3b as a picture. It is equipped with precision positioning devices and controlled by software written in LabVIEW. The sensor can be moved with the drives in all three axes of the Cartesian coordinate system. It can be rotated by $d_{r}$ in the *x*–*y* plane around the center of the sensor. Additionally, the height with respect to the target can be adjusted by $d_{z}$. The target is an electrically conductive sphere, which is connected to the ground potential. It is displayed in Figure 3a,b as a red ball. All used positioning components for the test bench are outlined in Table 1, together with the assignment to the individual axes. We used components with low positioning errors to ensure the high accuracy of the acquired ground truth distance. The linear positioning devices have accurate drives, with errors in the micrometer range and the rotary positioning device in the range of arc seconds. The position error for every axis is displayed in Table 1.

The test bench is used to generate training data and to verify the sensor algorithms; it operates in a per-sample mode. The procedure to acquire a new sample from the test bench is described in Algorithm 1. The acquisition process starts with a text-based input file, which is read by the LabVIEW control software. The input file is organized as a table, where each row contains position values for axis *x*, *y* and *z* in millimeters and for the rotational axis r in degrees. The LabVIEW control software parses the position values in each row into commands for the drives to move the sensor to a defined position. Once the position is reached, the LabVIEW software starts to acquire data from the sensor; namely, the CIC values and the sensor temperature. Additionally, the position value and a timestamp are saved. The resulting file contains the measurements as defined in the input text file. Since moving to a specified position depends on the length of the path, the acquired samples are not equidistant with respect to time. Additionally, an external temperature sensor is used to track environmental parameters and as a reference for the internal temperature sensor of the capacitive skin.
**Algorithm 1** Acquisition procedure to capture sensor samples at specified positions.1:Read *input text file*2:**for** number of rows in *input text file* **do**3:    Parse position values into commands4:    Move axes to position5:    **repeat**6:         Check Position Reached7:    **until** Position Reached8:    Read *CIC* and write to result file9:    Read *temperature* and write to result file10:  Read *position* and write to result file11:  Read *timestamp* and write to result file12:**end for**

### 2.4. Signal Processing Algorithms

Various algorithms are deployed on the sensor system using the MATLAB Embedded Coder. All signal processing algorithms are displayed in the flow chart in Figure 1 as green boxes. The first method in the processing chain receives the raw sensor readings RX0 to RX4 and the sensor temperature in degrees Celsius. *Temperature compensation* uses a linear model with slope $\vec{a_{\vartheta }}$ and offset $\vec{b_{\vartheta }}$:

(1) $${\vec{RX}}_{TC}=\vec{RX}-\left(\left(\vartheta -\vartheta_{R}\right)\;\cdot \;\vec{a_{\vartheta }}+\vec{b_{\vartheta }}\right).$$

These five-by-one vectors contain the temperature dependency for every receiving channel. The temperature coefficients are used to calculate the offset with respect to the reference temperature $\vartheta_{R}$. New sensor data indicated as vector $\vec{RX}$ are then compensated by applying the linear model. The temperature coefficients $\vec{a_{\vartheta }}$ and offset $\vec{b_{\vartheta }}$ are computed during the training time for the training data set. Sensor signal offsets are extracted by using the available ground truth for distances larger than 380 mm. Then, a linear model using least squares regression is constructed to map a temperature increase to a signal increase.

A second compensation module called drift compensation is implemented to account for both long-term signal drift and for differences in signal offset between individual sensors. This module is built as a linear compensation, defined as follows:

(2) $${\vec{RX}}_{C}={\vec{RX}}_{TC}-\left({\vec{RX}}_{SO}-{\vec{RX}}_{O}\right).$$

Given by the difference of the actual sensor offset ${\vec{RX}}_{SO}$ with respect to the known offsets ${\vec{RX}}_{O}$ during training, the sensor offset is compensated to ${\vec{RX}}_{C}$. This difference is then subtracted from the data sample, which has previously been temperature compensated. The process to extract the current sensor offset ${\vec{RX}}_{SO}$ needs to be performed when there is no obstacle close to the sensor. This can be done during the downtime of the sensor environment.

The neural network pre-processes the temperature and drift-compensated sample by scaling the signal through the mean and signal standard deviation obtained during training. This step is required to scale all signals in a comparable range before the inference is started. During inference, a new output of the target values distance and angle, denoted as *y*, is predicted by using the transfer function *f* of the neural network weights and biases w and the compensated input vector ${\vec{RX}}_{C}$:

(3) $$y=f(w,{\vec{RX}}_{C}).$$

For the optimization of the neural network, the mean squared error (MSE) loss function is used:

(4) $$MSE=\frac{1}{N}\;\cdot \;\sum_{n=1}^{N}{\left(y_{n}-y_{n}^{\prime }\right)}^{2}.$$

This function is minimized such that a low training error between the predicted value $y_{n}^{\prime }$ and target value $y_{n}$ is achieved. The target values $y_{n}$ are acquired in the sensor skin test bench. The parameter *N* is the number of samples to compute the metric.

For the data quality indication of sensor signals, a one-class SVM has been used. The idea of one-class SVMs is to classify samples into known and unknown instances. This binary classification scheme allows data quality indication based on the available training data, which is achieved by constructing a hyper plane that separates normal and abnormal data. This unsupervised learning problem learns the decision function f(x):

(5) $$f\left(x\right)=sgn\left(\sum_{i}\alpha_{i}k\left(x_{i},x\right)\right).$$

The parameters α are the trained coefficients of the SVM classifier, and $x_{i}$ represents the support vectors. The decision function evaluates to a positive value of +1 for samples that lie within the underlying distribution and to −1 for the complement [25]. The decision function is parameterized by the kernel function *k* given as a radial basis function kernel and the kernel scale parameter γ:

(6) $$k\left(x_{i},x\right)=e^{-\gamma ||x_{i}-x{\left|\right|}^{2}}.$$

The variable γ is used to control the smoothness of the decision function. It directly influences the number of data points used as support vectors. Before the data can be used for training and inference, a dedicated feature scaling block is introduced in the system architecture in Figure 1. The block receives temperature and drift-compensated samples for feature scaling. First, the inverse of new incoming data points is taken. This allows the SVM to focus more on values for far distances, as their values are typically low, while capacitive values for near distances are high. By inverting the incoming signals, this significance in magnitude is changed. To assess the performance of the proposed one-class SVM for data quality indication, we compare it with two baseline approaches:

1. A distance based method using k-NN search. We calculated the average inter-sample distance in the training data set to k nearest neighbors and used this as a threshold. For new samples, the existing training data are used to find the average distance to the k nearest training samples. If this distance exceeds the pre-computed threshold, it is treated as out-of-distribution.
2. A threshold based approach. We calculated the average signal levels at distances above 200 mm and the standard deviation of these signals. The average signal and twice its standard deviation are used to evaluate in and out-of-distribution samples. Samples below the calculated threshold are treated as low data quality measurements.

An advantage of SVMs is the fact that they compress the model for classification, unlike other algorithms such as k-nearest neighbors. Secondly, support vector methods have a known computational complexity [26]. Both aspects are important when methods are deployed on resource-constrained devices. The developed test-bench works on a per-sample basis; it is not capable of capturing equidistant time series. Due to this fact, algorithms targeted towards time series analysis are not part of this work. The evaluated methods detect outliers that result in low data quality. The information about data quality from the SVM can be directly related to the output predictions. This can be done by the sensor, as it can raise an error on the emergency output, or by transmitting a special error message through the UART communication interface.

### 2.5. Data Sets

Different data sets were acquired to generate training data and to verify the developed algorithms. An overview of the used data sets is given in Table 2. The input data for the positioning system were predefined and kept the same for all data sets. Input data were specified such that the sensor moved away from and towards the target at randomized positions and angles. However, the input data were sorted such that the sensor would move in one direction with the endpoints of 0 and 390 mm and vice versa multiple times. As the detection range of the smart capacitive sensor skin was limited to 200 mm, fewer points were acquired for the far distance. These values were later used to evaluate the binary classification performance of data quality indication algorithms. All data sets were acquired over various days to ensure different environmental conditions and thus varying data distributions.

Data set number 1 was used for the training and validation of both the neural network and data quality indication algorithms. The data sets with IDs 2 to 8 were used to test the algorithm performance, especially data quality indication based on out-of-distribution detection and the prediction accuracy of distances and angles. The data in data set 1 were filtered to exclude measurements above the 200 mm detection range. The filtered data were shuffled randomly and split into training, test and validation parts. Training data were used to minimize the loss function during training, validation data were used to evaluate the loss during training, and test data were held behind to measure the overall algorithm performance after training.

## 3. Results

The sensor, as well as all developed signal processing algorithms, were tested with data acquired from the test bench, as displayed in Figure 3. According to the block diagram in Figure 1, the results for the individual signal processing blocks are presented below.

### 3.1. Temperature Compensation

The results for the temperature compensation block are summarized in Table 3. The temperature coefficients $a_{\vartheta }$, given as CIC signals per degree Celsius, are given before and after compensation as well as their relative change in percentage for the individual receiving channels. The values are calculated by extracting the coefficients per channel for every data set. To aggregate the information, the average is calculated over all data sets for every RX channel.

### 3.2. Drift Compensation

**Figure 4:**
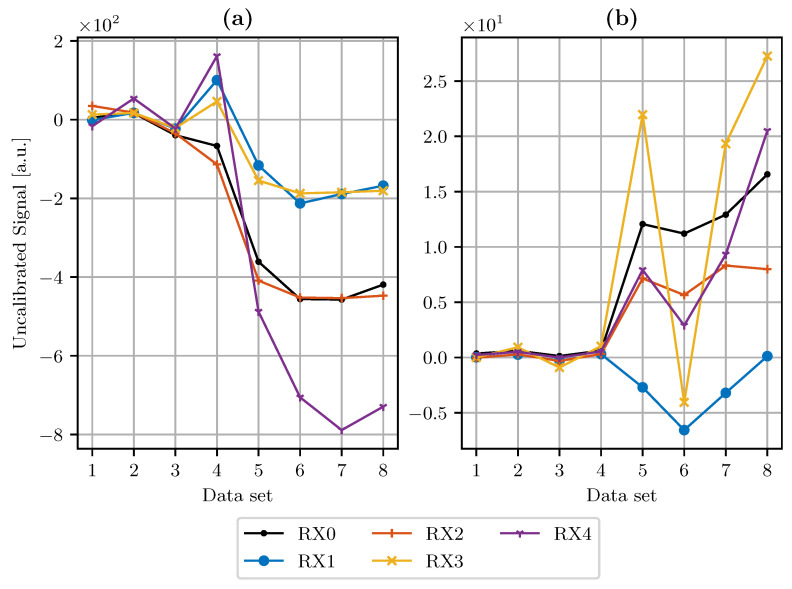
Sensor signal offset of all data sets without drift compensation applied (**a**) and with drift compensation applied (**b**).

To evaluate the results of the drift compensation module, the signal offset at distances larger than 380 mm is extracted after temperature compensation. These values are compared to the results after Equation (2) was applied to the signals. The results are shown in Figure 4. The signal offsets displayed in Figure 4a are deviations from the reference offset value for all data sets. Figure 4b shows this difference after drift compensation was applied.

### 3.3. Binary Classification Results

Results for binary classification are determined by creating labels for in and out-of-distribution samples. We defined in-distribution samples during training as data points with a distance of 200 mm and less. Out-of-distribution samples, which are outliers considered as having low data quality, were all samples acquired at distances larger than 200 mm. As metrics, we used F1-Score, precision, and recall. The scores were calculated for all data sets, and the results are given in Table 4.

**Figure 5:**
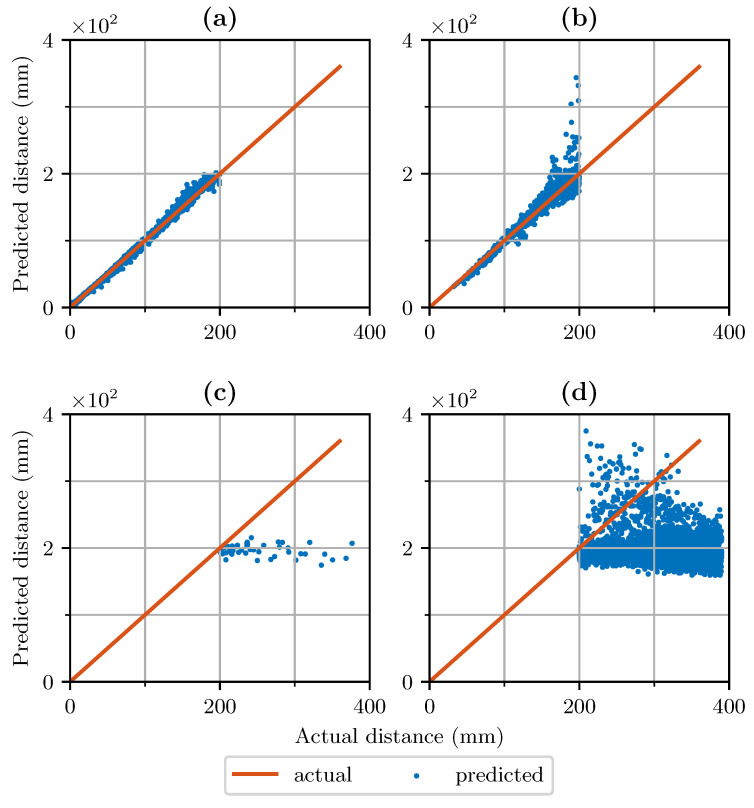
Results of distance prediction with data quality indication of data set 2 based on SVM-829. (**a**) True positive predictions with good data quality, (**b**) false negative predictions. (**c**) false positive predictions, (**d**) true negative predictions with low data quality.

For data quality indication, two variants of a one-class SVM were developed: one with 827 support vectors and one with 5763 support vectors. For comparison, the distance-based k-NN classifier was trained as well as a threshold-based algorithm. Furthermore, Figure 5 gives an overview of the effect of data quality monitoring based on data set 2 and SVM-827. The binary classification results are mapped to the distance output of the sensor.

### 3.4. Prediction of Distance and Angle with Data Quality Indication

For the evaluation of the regression task, the mean absolute error (MAE) and coefficient of determination $R^{2}$ are used. The results are shown in Table 5 for distances in millimeters and in Table 6 for the angle in degrees. Every metric is calculated with data quality based on the four chosen OOD algorithms. The last columns are the results for distance and angle prediction without data quality indication. To evaluate the sensor outputs with data quality, the decision functions of the OOD algorithms were evaluated for positive and negative classes. The metrics for angles were calculated in the interval between 10 and 350°. We observed that, close to 0 and 360°, the predicted value sometimes wrapped, which represents a small error in terms of the unit circle but a large error in terms of common regression metrics. Additionally, both tables have a row with the average metric over all data sets.

The results were determined based on a neural network, trained in MATLAB for 700 epochs, using Bayesian regularization backpropagation for optimization. The network consisted of three hidden layers of sizes 16, 12 and 11 with tanh activation functions. The layer sizes were found by evaluating the MSE loss curves during training. As input data, the five-dimensional CIC vector was used, which was preprocessed according to Figure 1. The three-dimensional output vector *o* is defined as follows:

(7) $$o=\left(\begin{array}{c} Distance\\ d_{x}\\ d_{y} \end{array}\right)$$

It contains the distance to the target and Cartesian object coordinates $d_{x}$ and $d_{y}$ of the target. The outputs are mapped from the last hidden layer using linear activation functions. To extract the angle φ from the Cartesian coordinates, the following equation is applied:

(8) $$\varphi =tan^{-1}\left(\frac{d_{y}}{d_{x}}\right)$$

### 3.5. Computational Cost

The usage of code and data memory was measured in bytes by inspecting IAR Embedded Workbench linker map file, the results are given in Table 7 in bytes. The algorithms were deployed on the sensor system as C-code libraries generated with MATLAB embedded coder. Both algorithms were built using 32-bit floating-point numbers for numerical operations. Execution time was measured in milliseconds with an oscilloscope using output pins of the microcontroller. The pins were pulled high when entering the function under test and pulled low once the execution finished.

### 3.6. Evaluation Metrics Dependent on Computational Complexity

**Figure 6:**
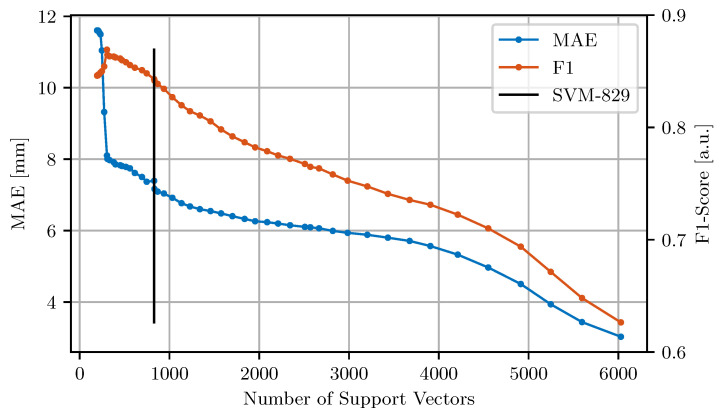
Computational complexity measured by number of support vectors and its influence on regression and binary classification metrics.

The dependency of regression and binary classification metrics was analyzed with respect to computational complexity. On the abscissa in Figure 6, the number of support vectors is displayed. The figure has two ordinate axes: the mean approximation error on the left side and F1-score on the right. The black line shows the position of SVM-829 within this study. The models with different numbers of support vectors were created by conducting a parameter study over the kernel variable γ. The model SVM-829 suits the constraints of the smart capacitive sensor skin best. We found that the model with more support vectors is too costly, taking too much time to run within 5 ms.

## 4. Discussion

The chosen linear model for temperature compensation reduces the temperature coefficients $\vec{\alpha_{\vartheta }}$ by an average of 83.5% over all data sets and RX channels, making the system more stable against temperature changes. Figure 4a shows the sensor signal offset per data set after temperature compensation was applied. We observe that data sets which are closer in time to the training data set show a smaller offset deviation. Remaining differences to the reference offset are compensated by the drift compensation module. This worked well for all data sets. However, we still observe a growing deviation from the reference offset in Figure 4b, which is set to zero as the data set number increases. Still, all compensated offsets remain in the range between −6 and 27. The uncompensated offsets range between −788 and 916. Thus, the fluctuation range is reduced by one order of magnitude compared to an uncompensated sensor offset. The compensation modules can be integrated in the system at low effort directly in software. Comparable approaches have used a reference electrode to account for temperature effects [8].

The results of the regression problem of distance prediction are aggregated in Table 5. For distance prediction, a mean absolute error over all data sets without data quality indication of 11.64 mm can be reported, with a fluctuation range between 3.2 and 14.7 mm. The mean error is reduced to 7.4 mm if the data quality indication based on SVM-829 is taken into account. The more complex SVM-5763 variant with a higher number of support vectors reduces the average error to 2.4 mm. If the threshold is used for data quality indication, the mean absolute error of the capacitive sensor skin is reduced to 4.5 mm; for k-NN, it is reduced to 10.4 mm. The out-of-distribution detection algorithm SVM-829 reduces the overall error for the prediction of distances between 0 and 200 mm by a factor of 1.6. The coefficient of determination $R^{2}$ also increases if data quality is considered. Considering data quality indication based on SVM-829, the average error is almost halved and the fluctuation range is reduced by a factor of two. The angle prediction is calculated in the interval between 10 and 350°. If data quality is not taken into account, the average angular error is 15.6°, with a minimum error of 3.3° and a maximum error of 20.1°. If the data quality of incoming RX measurements based on SVM-829 is considered, the average angular error is reduced to 14.1°, the minimum value is 6.9° and the maximum value is 25.3°. The second SVM variant with 5763 support vectors reduces the overall angular error to 7.6° degree, while the threshold reduces it to 12.5° degrees. For the distance-based k-NN approach, we can report an average approximation error of 15.6 degrees.

The evaluation of the binary classification problem was conducted by treating sensor readings with a distance above 200 mm as low data quality, out-of-distribution samples. The results are summarized in Table 4. The average F1-Score of SVM-829 for all data sets is 0.84. The average precision of the classifier is 0.84, while the recall is 0.89. The OOD algorithms SVM-5763, threshold and k-NN achieve F1 scores of 0.59, 0.66 and 0.85, respectively. It can be observed that the more complex SVM and the threshold-based classifier lack generalization. Both achieved precision scores close to one, meaning that the samples classified as in-distribution were part of this class. However, both algorithms have a significantly lower recall score compared to SVM-829 and k-NN, which means that both failed to classify relevant samples into the correct class. Precision in this case reports the overall performance of classifying samples with less than 200 mm as such samples. We observe that the SVM-829 does misclassify some samples as good data quality that should be labeled as bad data quality instead. However, based on the acquired data, we observed that these data points still generate valid distance predictions. For data quality indication, binary classification metrics are an important measure but also the output of the regression algorithm. This is also highlighted in Figure 5a. For distances between 0 and 200 mm, all samples in terms of binary classification should be identified as having high data quality. However, as can be seen in Figure 5b, the classifier filtered values that significantly deviated from the target value but also values close to the target value. Moreover, distances above 200 mm, were falsely classified as in-distribution, as shown in Figure 5c. These results can also be observed in Table 4. For data set number 2, the precision was 0.97. The recall score of this data set was 0.76, meaning it detected not all true positive samples compared to the number it should have detected.

The computational cost was quantified by measuring the code size and execution time. The available time budget was 5 milliseconds for one processing step, which consisted of the following parts: reading the sensor MGC3130, processing a new sample trough temperature and drift compensation, predicting the distance and angle and quantifying the data quality by evaluating the SVM decision function. The most important aspect in terms of computational cost is the execution time. We measured an execution time of 0.14 milliseconds for the neural network with three hidden layers. Evaluating the SVM decision function takes 1.54 milliseconds; the computationally heavy tasks mainly involve predicting the input samples in the feature space. The larger execution time corresponds with the increased memory demand of the SVM compared to the neural network. Execution time and memory usage could be further reduced by using fixed point numbers for the models.

The design of a valuable data quality indication algorithm requires a balance between the impact on the sensor signal and binary classification metrics. If the algorithm runs on a resource-constrained system, the computational cost has to be considered as well. This area of conflict is visualized in Table 4, Table 5 and Table 6. If only binary classification metrics were considered, the k-NN based algorithm performed best on average over all data sets. However, the distance-based OOD variant has the least positive effect on the sensor output and additionally requires all training data points to be stored for inference. This makes k-nearest neighbors infeasible for an embedded data quality indication algorithm. The more complex SVM variant with 5763 support vectors and the threshold-based approach both perform better in improving the overall sensor output but lack generalization in terms of binary classification metrics. This issue is also visualized in Figure 6. We observe a decreasing average approximation error with more complex SVMs, but also a lower average F1-Score due to overfitting. Secondly, as the complexity increases, the accuracy in terms of binary classification metrics also decreases. This trade-off must be balanced individually for an application. For the smart capacitive sensor skin, the constrained computational complexity and high classification error combined with low mean average regression error had to be leveled. Other applications might have more computational power or could allow a higher number of false alarms. In addition, the large number of learned support vectors is computationally too costly for deployment on a small microcontroller. The threshold-based variant does not have this disadvantage but produces too many false alarms as well. Finally, the best solution was the out-of-distribution detection algorithm SVM-829. With this algorithm, the quality of the data could be rated most efficiently. The overall error for the prediction of distances between 0 and 200 mm was improved by a factor of 1.6 by considering the data quality indicator.

## 5. Conclusions

The proposed system as well as the developed algorithms performed well within the specified requirements. Temperature compensation reduces the overall influence of temperature changes on the system by 83. The effect of temperature gradients in capacitive sensors has been studied by Hoffmann et al. [8]. Their approach was to use an additional electrode to compensate temperature effects, which requires more space on the sensor and adds complexity to the overall system design. Our approach can be implemented in software. The prediction errors for distance and angle are in line with other studies. For the distance prediction, the approach of Poeppel et al. [9] has been outperformed for distances with less than 200 mm. They reported an average error of 20.7 mm with a detection range of 350 mm. Erickson et al. reported a position error of 11 mm for ranges between 0 and 100 mm; at 200 mm, their error increased up to 56 mm [10]. Our approach reached an average error of 11.6 mm without data quality indication and 7.4 mm with data quality indication based on SVM-827 across a 200 mm detection range. Additionally, Erickson et al. reported an angular error of 6.2° between 0° and 30° and 11.0° between 30° and 45°. We achieved an angular error between 10° and 350° of 15.59°. If data quality is considered based on SVM-827, the mean angular error is reduced to 14.14°. All data processing algorithms for distance and angle estimation can be processed in real time. The proposed support vector machine method to monitor signal quality was deployed on the sensor system. It improved the overall system performance and detected data quality issues successfully. Data quality indication is thus a valuable tool to enhance the safety of human–robot interaction scenarios. It reduces the overall number of erroneous predictions and increases system reliability. Additionally, we demonstrated the real-time capabilities of the methods based on measurements. The more complex SVM variant with 5763 support vectors has a very positive impact on the overall sensor output. However, if evaluated in terms of binary classification metrics, too many false alarms would make a sensor system unusable. On the other hand, an algorithm such as k-NN has good binary classification scores, comparable to SVM-827, but very little impact on data quality indication.

## 6. Outlook

The presented system and algorithms do not consider the time dependency of input signals, neither for the neural network nor for the out-of-distribution detection algorithm. We will evaluate more complex data-driven approaches such as long short-term memory (LSTM) or recurrent neural networks (RNN) in future. However, involving the time dimension in all predictions also increases the system latency, which might not be desirable for human–robot interaction use cases. Secondly, knowledge-based approaches could also be investigated to perform data quality indication on the sensor level. Additionally, not all errors with respect to sensor performance result from OOD samples. Different methods to account for additional data quality-related parameters are necessary. The method for data quality indication and especially the signal processing pipeline with a dedicated quality of sensing module proposed in Figure 1 is not limited to human–robot interaction scenarios and can be adapted to various use cases to enhance the reliability of sensors and sensor systems.

## Figures and Tables

**Table 1:** Test bench parts, part specification and axis assignment.

Part	Specification	Axis
Owis LTM120M-390-HSM	390 ± 25 × 10^−3^ mm	*x*
Owis LIMES90-100-HDS	200 ± 16 × 10^−3^ mm	*y*
Owis LIMES90-100-HDS	200 ± 16 × 10^−3^ mm	*z*
Owis DMT65-DM6-HDS	0.360° ± 0.02°	*r*
LabVIEW	Version 2018	-

**Table 2:** Properties of used data sets.

Data Set	Total No. of Instances	No. of Instances > 200 mm
1	33,116	9122
2	32,557	8632
3	29,532	7609
4	30,041	8118
5	30,256	8334
6	29,575	7652
7	30,006	8083
8	29,934	8011
train	16,776	-
test	3594	-
validation	3595	-

**Table 3:** Temperature coefficient with and without temperature compensation and improvement in percent.

CIC-Channel	Not Compensated $a_{\vartheta }$ $\left[\frac{CIC}{{}^{\circ }C}\right]$	Compensated $a_{\vartheta }$ $\left[\frac{CIC}{{}^{\circ }C}\right]$	(%)
RX0	56.14	10.26	84.11
RX1	56.43	9.78	79.03
RX2	66.20	9.03	84.01
RX3	229.51	19.49	92.51
RX4	58.34	11.05	78.26

**Table 4:** Binary classification results.

Data Set	QoS Algorithm	SVM-829			SVM-5763			Threshold			k-NN		
	Metric	F1	Precision	Recall	F1	Precision	Recall	F1	Precision	Recall	F1	Precision	Recall
1		0.90	0.84	0.96	0.63	1.00	0.46	0.70	1.00	0.54	0.87	0.77	0.99
2		0.86	0.97	0.76	0.51	1.00	0.34	0.49	1.00	0.32	0.85	0.80	0.92
3		0.89	0.81	0.99	0.76	1.00	0.62	0.75	1.00	0.60	0.87	0.76	1.00
4		0.64	1.00	0.47	0.01	1.00	0.00	0.14	1.00	0.08	0.78	0.71	0.87
5		0.84	0.73	1.00	0.73	1.00	0.57	0.82	0.99	0.70	0.84	0.73	1.00
6		0.91	0.87	0.95	0.65	1.00	0.48	0.63	1.00	0.45	0.89	0.80	0.99
7		0.86	0.75	0.99	0.75	1.00	0.60	0.85	0.84	0.86	0.85	0.74	1.00
8		0.85	0.75	0.99	0.70	1.00	0.54	0.87	0.91	0.84	0.85	0.73	1.00
Average		0.84	0.84	0.89	0.59	1.00	0.45	0.66	0.97	0.55	0.85	0.75	0.97

**Table 5:** Prediction of distance with data quality indication algorithms and without data quality indication.

Data Set	QoS Algorithm	SVM-829		SVM-5763		Threshold		k-NN		None	
	Metric	MAE	R${}^{2}$	MAE	R${}^{2}$	MAE	R${}^{2}$	MAE	R${}^{2}$	MAE	R${}^{2}$
1		4.35	0.99	1.44	1.00	1.88	0.99	4.44	0.99	4.48	0.99
2		2.12	1.00	0.88	1.00	0.80	1.00	2.87	0.99	3.21	0.99
3		3.83	0.99	1.57	1.00	1.57	0.99	3.88	0.99	3.90	0.99
4		7.60	0.86	1.07	0.77	1.72	0.79	33.19	−0.60	39.95	−0.64
5		9.30	0.94	3.12	0.98	5.10	0.96	9.30	0.94	9.30	0.94
6		5.58	0.98	2.15	0.99	1.72	0.99	5.81	0.98	5.92	0.98
7		11.67	0.91	4.50	0.96	11.68	0.90	11.64	0.91	11.64	0.91
8		14.71	0.87	4.44	0.96	11.97	0.89	14.72	0.87	14.72	0.87
Average		7.40	0.94	2.40	0.96	4.55	0.94	10.73	0.76	11.64	0.75

**Table 6:** Prediction of angle with data quality indication algorithms and without data quality indication.

Data Set	QoS Algorithm	SVM-829		SVM-5763		Threshold		k-NN		None	
	Metric	MAE	R${}^{2}$	MAE	R${}^{2}$	MAE	R${}^{2}$	MAE	R${}^{2}$	MAE	R${}^{2}$
1		9.54	0.94	5.53	0.97	7.13	0.96	9.72	0.94	9.80	0.94
2		6.94	0.93	5.87	0.94	9.36	0.85	8.49	0.90	9.55	0.89
3		9.78	0.94	5.98	0.97	6.79	0.97	9.85	0.94	9.87	0.94
4		25.37	0.59	12.25	0.85	23.80	0.34	30.64	0.56	33.37	0.54
5		14.07	0.88	6.85	0.99	9.28	0.96	14.08	0.88	14.08	0.88
6		9.30	0.94	7.27	0.95	7.45	0.94	9.29	0.94	9.44	0.94
7		19.97	0.80	9.06	0.97	20.12	0.79	20.08	0.79	20.10	0.79
8		18.13	0.85	8.05	0.98	16.20	0.87	18.30	0.85	18.54	0.84
Average		14.14	0.86	7.61	0.95	12.52	0.83	15.05	0.85	15.59	0.84

**Table 7:** Computational cost and execution time. Memory given in bytes, time in milliseconds.

	Neural Network	One-Class SVM
Code memory	2868	20,900
Data memory	-	43,108
Execution Time	0.14	1.54

## Data Availability

The data underlying this article will be shared on reasonable request from the corresponding author.
